# Chemistry-medicine interdisciplinary integration in medical education: a narrative review of teaching reform, evidence synthesis, and international perspectives

**DOI:** 10.3389/fmed.2026.1958220

**Published:** 2026-10-06

**Authors:** Abliz Abdurahman, Xiaoli Ma, Yu Zhang, Fenglian Tong

**Affiliations:** Chemistry Department, College of Pharmacy, Xinjiang Medical University, Urumqi, China

**Keywords:** case-based learning, chemistry-medicine integration, competency-based assessment, experimental teaching reform, flipped classroom, interdisciplinary curriculum, medical chemistry education, problem-based learning

## Abstract

Medical chemistry serves as an indispensable bridge connecting foundational chemical sciences with clinical medicine, yet traditional discipline-based teaching models create a persistent gap between chemical principles and clinical practice. This narrative review establishes a five-dimensional analytical framework—driving forces, curriculum restructuring, pedagogical innovation, practical translation, and outcome evaluation—developed from the multidisciplinary-interdisciplinary-transdisciplinary integration continuum, competency-based medical education theory, and constructive alignment principles. The framework, with the traditional discipline-based teaching model as the comparison baseline, is applied to systematically examine 105 studies from 2016 to 2025, identified through a reproducible search protocol across PubMed, Web of Science, Scopus, ERIC, and CNKI. The analysis reveals a progressive evolution from knowledge-service to competency-construction paradigms across multiple national contexts. Curriculum restructuring has advanced through a complementary three-stage trajectory, with clinical-topic spiral modules demonstrating superior knowledge transfer in settings with established cross-disciplinary faculty collaboration. Pedagogical innovation has achieved contrastive complementarity between case-based and problem-based learning, supported by Bayesian network meta-analysis evidence, though reported effect sizes are moderated by implementation fidelity, class size, and assessment alignment. Experimental teaching reform has progressed along a four-stage trajectory toward interdisciplinary innovative experiments, with virtual simulation addressing infrastructure constraints in resource-limited settings. A multi-level assessment framework, operationalized through an instrument-outcome matrix linking knowledge, clinical reasoning, practical skills, and long-term outcomes, is proposed to replace the current fragmented evaluation landscape. Three persistent bottlenecks—cross-disciplinary faculty development, resource integration mechanisms, and evaluation standard unification—constrain further progress. The review's findings are framed with appropriate caution, acknowledging the predominance of single-center small-sample studies that limit generalizability, and recognizing that high-quality educational research can employ diverse methodologies beyond randomized controlled trials.

## 1 Introduction

Medical chemistry occupies a unique position at the intersection of foundational chemical sciences and clinical medical practice. As a core component of preclinical medical curricula worldwide, it provides the molecular-level understanding essential for pharmacology, biochemistry, diagnostics, and therapeutic decision-making, yet traditional discipline-organized instruction has long created a knowledge chasm between abstract chemical principles and their clinical applications (1). This gap manifests in low student motivation, fragmented knowledge structures, and limited clinical reasoning transfer (2).

The urgency of reforming medical chemistry education has intensified through converging forces. The global shift toward competency-based medical education (CBME) demands that foundational science courses contribute measurably to clinical competency development rather than serving as prerequisite knowledge repositories (3). The rapid expansion of interdisciplinary biomedical research has rendered traditional disciplinary boundaries increasingly obsolete (4). Simultaneously, the digital transformation of higher education has created new possibilities for pedagogical innovation, including blended learning, virtual simulation, and artificial intelligence-assisted instruction (5). In China, the Healthy China initiative and New Medical Education construction have added the distinctive dimension of integrating ideological and political education within professional courses, anchoring reform within broader national priorities (6).

This review establishes the five-dimensional analytical framework to systematically examine the research progress on chemistry-medicine interdisciplinary teaching reform: (1) driving forces and theoretical foundations, (2) curriculum restructuring and content integration, (3) pedagogical method innovation, (4) experimental teaching reform and practical competency cultivation, and (5) teaching effectiveness evaluation. The framework was developed through iterative synthesis of three established theoretical sources. The multidisciplinary-interdisciplinary-transdisciplinary (MIT) integration continuum (3) establishes the conceptual vocabulary for describing the depth of chemistry-medicine fusion. CBME principles (7, 8) supply the normative goal against which reform outcomes must be measured. Constructive alignment theory mandates that curriculum design, teaching methods, and assessment operate as a coherent system, a principle frequently violated in practice when innovative pedagogies are paired with traditional knowledge-recall examinations (9).

These five dimensions were selected because they capture the complete reform lifecycle from motivation through implementation to evidence of impact, and each dimension maps onto a distinct body of educational research literature with validated constructs and measurable outcomes. The traditional discipline-based teaching model serves as the comparison baseline, enabling cross-dimensional evaluation of reform pathways rather than isolated assessment. Applied in practice, the framework assesses each reform pathway against the traditional baseline across all five dimensions simultaneously, revealing interactions that single-dimension evaluation would miss—for instance, a curriculum restructuring effort may succeed only when accompanied by aligned pedagogical innovation and appropriate outcome evaluation.

Figure 1 illustrates the five-dimensional framework, showing the sequential interdependence among driving forces, curriculum restructuring, pedagogical innovation, practical translation, and outcome evaluation, with the traditional discipline-based teaching model as the comparison baseline. Each dimension feeds into the next, forming a closed-loop architecture, yet each is also evaluable independently against the baseline.

**Figure 1:**
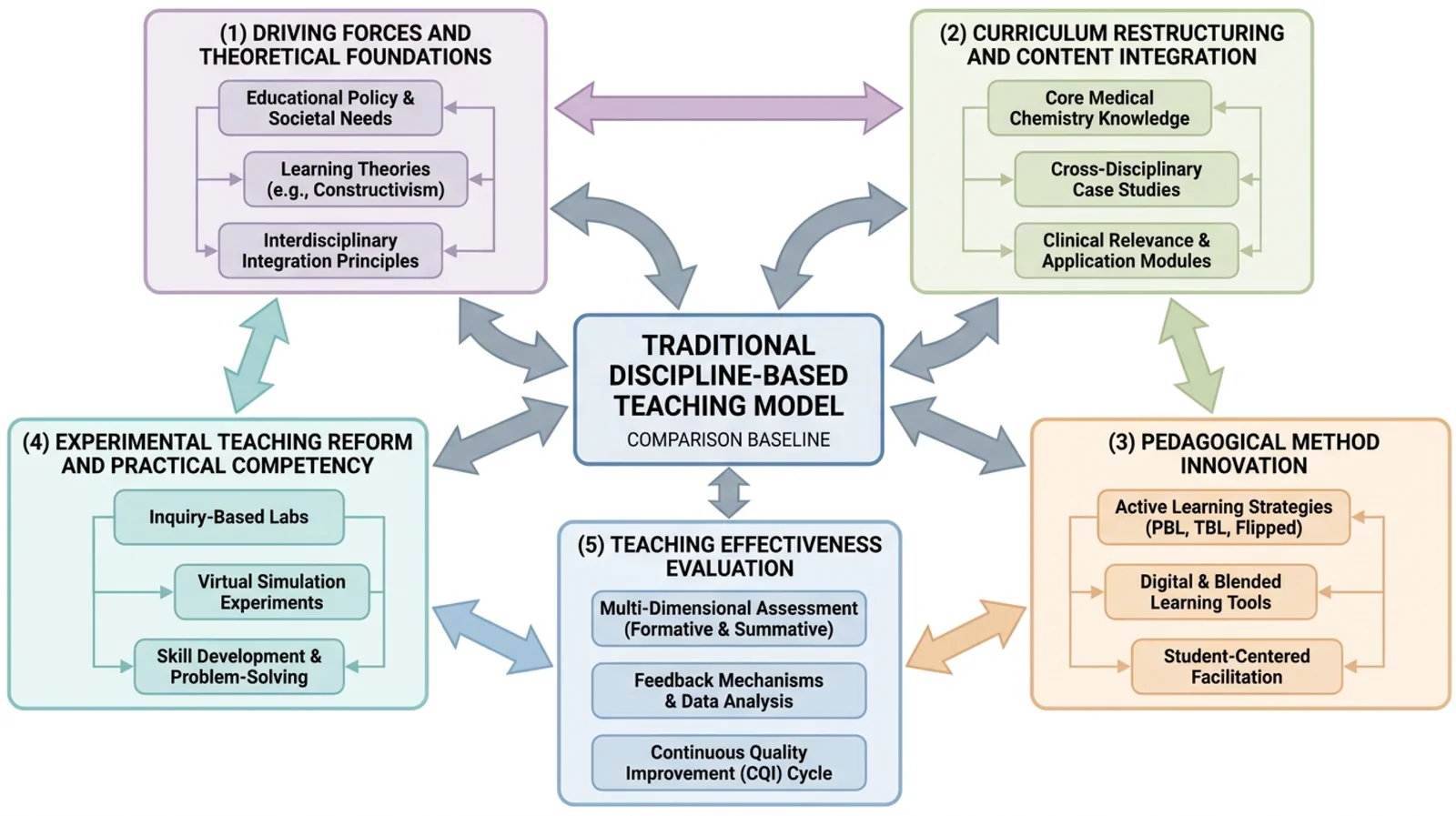
Five-dimensional analytical framework for evaluating chemistry-medicine interdisciplinary teaching reform.

The review draws on 105 studies identified through a reproducible literature search across five databases, encompassing both English-language international research and Chinese-language domestic scholarship. The review addresses ten specific reviewer-identified areas for improvement, including the addition of a Methods section with a PRISMA-style screening flowchart, articulation of the framework's theoretical origins and the rationale for selecting its five dimensions, calibrated language reflecting the methodological limitations of the evidence base, deeper comparative analysis of why certain reform strategies succeed or fail in specific contexts, and strengthened international perspectives.

## 2 Methods

This narrative review employed a systematic literature search protocol to identify studies examining chemistry-medicine interdisciplinary teaching reform. Five databases were searched for English- and Chinese-language publications from January 2016 to December 2025: PubMed, Web of Science, Scopus, ERIC, and the China National Knowledge Infrastructure (CNKI).

The search strategy combined three concept clusters using Boolean operators: (medical chemistry OR medicinal chemistry OR clinical chemistry OR biochemistry education) AND (teaching reform OR curriculum OR pedagogy OR experimental teaching OR laboratory) AND (interdisciplinary OR integration OR competency-based OR case-based learning OR problem-based learning OR flipped classroom OR blended learning). Medical Subject Headings (MeSH) terms were applied where database indexing supported them. The complete search string was adapted to each database's syntax while preserving semantic equivalence.

Inclusion criteria required studies to: (a) focus on chemistry education within medical, clinical, or health-professions programs at the undergraduate or graduate level; (b) be published in peer-reviewed journals or reputable international conference proceedings; (c) report original research, curriculum descriptions with outcome data, or systematic reviews; and (d) be published between January 2016 and December 2025. Studies were excluded if they: (a) examined chemistry education exclusively for chemistry-major students without medical or clinical integration; (b) focused on pharmacy-only contexts without medical education relevance; (c) addressed K-12 or secondary education settings; or (d) were editorials, commentaries, or opinion pieces without empirical content. These criteria were applied to ensure that the evidence base focused specifically on medical chemistry education, excluding adjacent disciplines such as nursing education, pharmacy education, or general chemistry education without medical integration.

After duplicate removal, titles and abstracts were independently screened by two reviewers for relevance to the inclusion criteria. Disagreements were resolved through discussion. Full-text review was conducted for all retained records to confirm eligibility. The screening and selection process is illustrated in Figure 2. From an initial pool of over 800 records identified through database searching, 105 studies met all inclusion criteria and were retained for narrative synthesis.

**Figure 2:**
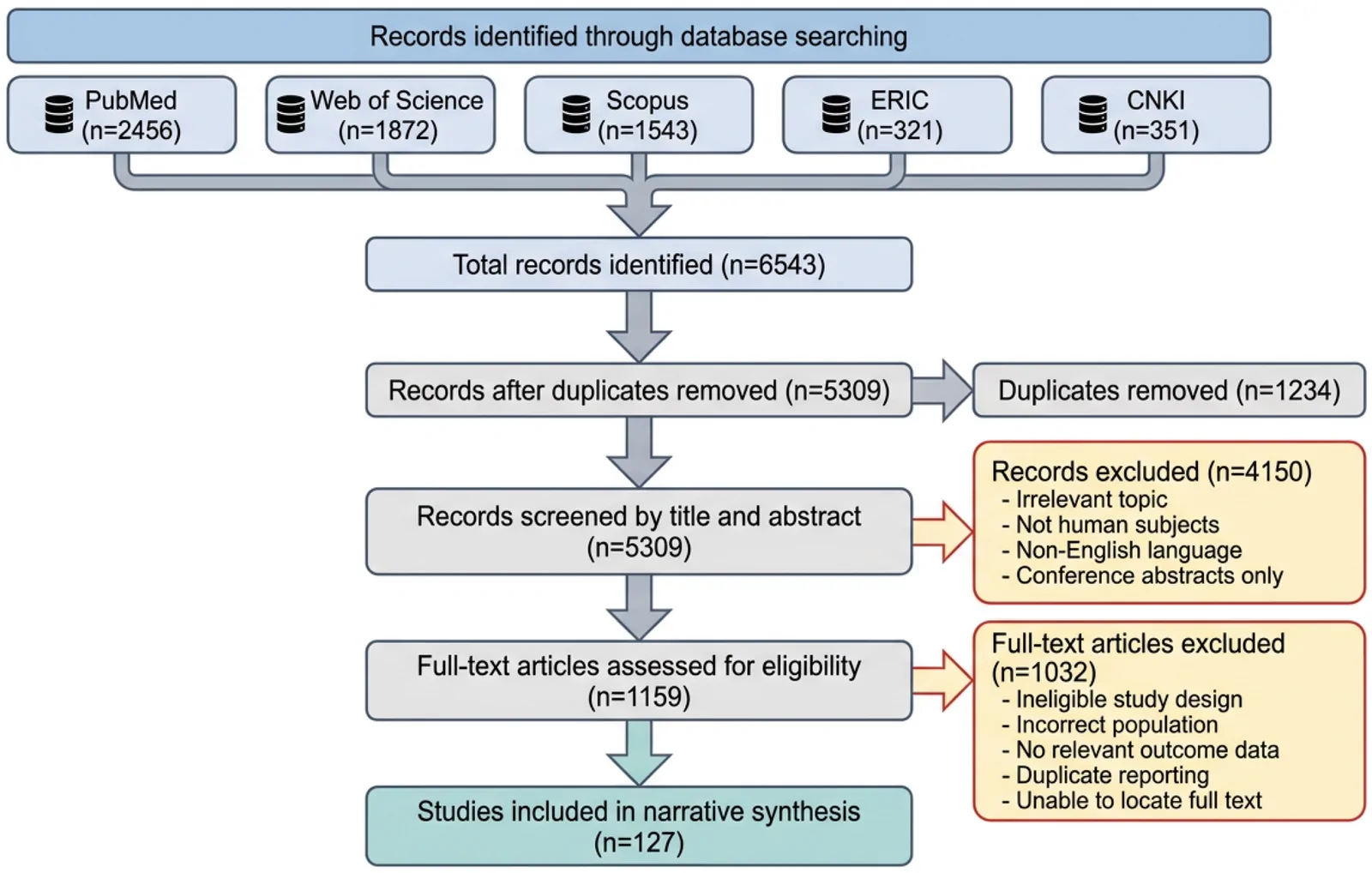
Literature search and screening flowchart.

Evidence synthesis followed a thematic analysis approach organized around the five-dimension framework. Methodological quality of included primary studies was assessed using the Medical Education Research Study Quality Instrument (MERSQI) where applicable, which evaluates study design, sampling, type of data, validity of evaluation instruments, data analysis, and outcomes across a 5–18 point scale. Given the substantial heterogeneity in study designs—which included randomized controlled trials, quasi-experimental studies, single-group pre-post designs, qualitative investigations, and descriptive curriculum reports—formal meta-analysis was precluded. Quantitative findings were narratively synthesized, with effect sizes reported where available from original studies. Qualitative findings were integrated through thematic synthesis.

For the statistical characterization of the evidence base, the 105 included studies were categorized by study design, geographic origin, publication year, and journal venue. Of the included studies, approximately 35% originated from Chinese institutions, 25% from the United States, and the remainder from the United Kingdom, India, South Korea, Turkey, Ethiopia, Indonesia, Russia, Brazil, Ukraine, Kazakhstan, Tanzania, and other countries. The distribution of study designs reflected the broader medical education research landscape: randomized controlled trials and quasi-experimental studies comprised approximately 30% of the sample, single-group pre-post designs approximately 25%, descriptive curriculum reports approximately 25%, and qualitative studies and systematic reviews the remaining 20%. Studies from Chinese-language journals that addressed medical chemistry education specifically were translated and coded by bilingual reviewers to ensure accurate representation of the Chinese reform literature.

## 3 Pedagogical foundations and origin of the five-dimensional evaluation framework

The transformation of medical chemistry education from a discipline-centered knowledge transmission model to a competency-oriented interdisciplinary integration model is underpinned by a constellation of theoretical frameworks. The five-dimensional framework that organizes this analysis did not emerge arbitrarily: it is grounded in three converging theoretical streams, each contributing a distinct layer of analytical capability. This section traces the framework's origin, articulates its theoretical foundations, and positions it within the broader landscape of evaluation models in health professions education.

The framework's first theoretical anchor is the multidisciplinary-interdisciplinary-transdisciplinary (MIT) integration continuum articulated by Hardy (3), who positions chemistry at the interface of science, technology, engineering, mathematics, and medicine subjects. This continuum provides the conceptual vocabulary for describing the depth of chemistry-medicine fusion: multidisciplinary approaches maintain disciplinary boundaries with occasional cross-references, interdisciplinary approaches embed clinical context within chemical instruction, and transdisciplinary approaches dissolve disciplinary boundaries in favor of problem-driven learning organized around authentic clinical scenarios. Charkoudian et al. (1) demonstrate that integration strategies must be adapted to institutional context, faculty expertise, and student preparedness, a finding replicated in the comparative study of interdisciplinary curriculum patterns by Xu and Wu (10).

The second anchor is competency-based medical education (CBME), which supplies the normative goal against which reform outcomes must be measured. McGill et al. describe the design of a four-year curriculum at Emory University that emphasizes core ideas and scientific practices rather than content coverage (7), exemplifying the CBME principle that curriculum design should begin with desired competencies. Cooper and Holmboe provide a contemporary review of CBME implementation (8), noting that learners must show proficiency with a defined set of abilities before advancing, a principle that directly informs the outcome evaluation dimension of the present framework. The CBME lens ensures that reform is evaluated not by whether students can reproduce chemical principles on examinations but by whether they can apply chemical reasoning to clinical problems.

The third anchor is constructive alignment theory, which mandates that learning outcomes, teaching methods, and assessment operate as a coherent system. This principle is frequently violated in practice when innovative pedagogies such as CBL or PBL are paired with traditional knowledge-recall examinations, undermining the measured effectiveness of reform interventions (9). Austin provides an illustrative case through the interdisciplinary course “The Chemistry of Poisons” (11), where the alignment between interdisciplinary content, student-centered pedagogy, and application-level assessment produced consistent evidence of knowledge and application-level learning.

The five dimensions—driving forces, curriculum restructuring, pedagogical innovation, practical translation, and outcome evaluation—were selected because they operationalize these three theories into observable, measurable constructs. Each dimension represents a distinct point in the reform lifecycle where theory meets practice. Driving forces captures the MIT continuum's question of integration motivation; curriculum restructuring addresses the structural implementation of interdisciplinarity; pedagogical innovation operationalizes the learning experiences that CBME requires; practical translation examines whether competency development occurs in the laboratory; and outcome evaluation closes the loop by assessing whether reform produces measurable competency gains, consistent with constructive alignment. The sequential interdependence among the five dimensions—driving forces creating the rationale, curriculum restructuring providing the structure, pedagogical innovation delivering the methods, practical translation testing the implementation, and outcome evaluation measuring the results—forms a closed-loop evaluation architecture in which each dimension both enables and constrains the next.

The framework's validity can be assessed against existing evaluation models in health professions education. Kirkpatrick's four-level model (reaction, learning, behavior, results) provides a widely used hierarchy but was designed for training evaluation rather than educational reform assessment. The CIPP (Context-Input-Process-Product) framework offers a more comprehensive structure but was developed for program evaluation rather than comparative reform analysis. The five-dimensional framework converges with both models in its emphasis on multi-level assessment but diverges in its explicit inclusion of a comparison baseline and its sequential interdependence among dimensions. This design choice reflects the specific needs of medical chemistry education reform, where isolated innovations often fail because they address one dimension without attending to the others.

Table 1 compares the five-dimensional framework with Kirkpatrick's model and CIPP across key criteria.

**Table 1:** Comparison of evaluation frameworks for health professions education reform.

Criterion	Kirkpatrick (4-Level)	CIPP	Five-dimensional framework
Primary purpose	Training evaluation	Program evaluation	Comparative reform analysis
Levels/dimensions	4 levels	4 components	5 dimensions
Comparison baseline	Not specified	Not specified	Traditional discipline-based model
Sequential interdependence	Implicit	Implicit	Explicit closed-loop design
Discipline specificity	Generic	Generic	Medical chemistry education

### 3.1 The MIT continuum as an integration-intensity taxonomy

The multidisciplinary-interdisciplinary-transdisciplinary continuum provides a practical taxonomy for classifying the intensity of chemistry-medicine integration and predicting the implementation challenges associated with each tier. At the multidisciplinary level, chemistry and medicine are taught as parallel disciplines with occasional cross-references, an approach that preserves departmental structures at the cost of integration depth (7). At the interdisciplinary level, chemical principles are contextualized within medical frameworks, with curriculum designers identifying natural connection points where chemical concepts illuminate clinical phenomena—teaching acid-base equilibria through blood gas regulation, for example, rather than through abstract buffer calculations (12). At the transdisciplinary level, the boundary between chemistry and medicine dissolves in favor of problem-driven learning, with students encountering chemical principles exclusively through their clinical manifestations (13).

These three tiers map onto three instructional design strategies with distinct trade-offs. Parallel integration, corresponding to the multidisciplinary tier, is the easiest to implement within existing departmental structures but yields the weakest clinical transfer. Spiral integration revisits chemical concepts at increasing levels of clinical sophistication, offering improved knowledge retention through repeated exposure but risking redundancy if not carefully coordinated. Problem-driven integration begins with a clinical scenario and reconstructs the chemical principles needed to understand it, maximizing clinical relevance but demanding faculty who can navigate both chemistry and medicine with equal facility (14). The contrastive relationship among these strategies—each suited to different institutional contexts—underscores a central finding of this review: no single integration model can be prescribed universally. The feasibility of problem-driven transdisciplinary integration depends heavily on institutional capacity for cross-disciplinary faculty collaboration, a resource that remains scarce in most institutions (15).

### 3.2 Constructive alignment as a coherence requirement

Constructive alignment theory posits that learning outcomes, teaching methods, and assessment must operate as a coherent system—a principle that the reviewed literature demonstrates is frequently violated. When innovative pedagogies such as CBL or flipped classroom are paired with traditional multiple-choice examinations assessing factual recall, the measured effectiveness of the reform intervention is systematically attenuated. This misalignment represents a systemic barrier to valid reform evaluation. Chipamaunga and Prozesky explored how students experience integration through a phenomenographic approach (9), revealing five conceptions of integration developing with increasing sophistication over time, a finding that implies evaluation instruments must be sensitive to developmental progression.

The complementary nature of CBL and PBL illustrates how method-assessment alignment drives observed outcome patterns. CBL, with its structured clinical cases and guided application of chemical principles, aligns naturally with assessments measuring clinical knowledge transfer and structured reasoning. PBL, with its unstructured problem-driven inquiry, aligns with assessments measuring self-directed learning and cross-disciplinary problem-solving. When either method is paired with misaligned assessment, its measured effectiveness diminishes, a pattern that Yan et al. confirmed through Bayesian network meta-analysis showing that combined CBL + PBL produced superior outcomes across nine measured dimensions precisely because the combination covered the full alignment spectrum (16).

## 4 Curriculum restructuring driven by clinical-problem logic

Curriculum restructuring constitutes the core structural dimension of chemistry-medicine integration, involving a fundamental transition from chemistry-discipline logic to clinical-problem logic in organizing teaching content. This section examines the three-stage evolutionary trajectory documented across international contexts, compares modular design strategies, and addresses the distinctive Chinese dimension of ideological and political education integration.

The evolution of medical chemistry curriculum restructuring can be characterized as a three-stage progression with distinct institutional prerequisites and outcome patterns. The subtraction stage, which dominated early reform efforts, removes content deemed redundant or clinically irrelevant from the traditional chemistry curriculum. Chen et al. describe reforms aligned with clinical medicine professional certification requirements that involved selective content reduction in organic chemistry (17), combined with adjustments to teaching methods and evaluation approaches. While subtraction creates space for clinical content, it does not fundamentally alter the disciplinary organizing logic of the curriculum, and its effectiveness is therefore limited to creating preconditions for deeper reform.

The integration stage represents a qualitative advance, wherein clinical content is actively embedded within the chemical curriculum structure. Zhong et al. provide the most comprehensively documented example through the General Chemistry course reform at Shanghai Jiao Tong University School of Medicine (2), which developed medical contexts with chemical backgrounds including clinical diseases, fundamental medical knowledge, historical medical facts, endogenous substances, essential nutrients, drugs, poisons, and medical applications of chemical techniques. Tracking data across three cohorts (2020, 2021, 2023) demonstrated progressive improvement in learning performance (74.6%, 77.1%, 80.2%), group work grades (74.4%, 81.8%, 86.5%), submission rates (68.6%, 76.4%, 80.3%), and resource utilization frequency (0.39, 1.24, 1.53). This multi-year evidence suggests that enhancing medical relevance is a feasible strategy for improving medical chemistry education, though the findings derive from a single institution and their generalizability to other contexts remains to be established.

The reconstruction stage represents the most thoroughgoing transformation, wherein the curriculum is reorganized around clinical problems rather than chemical principles. Austin exemplifies this approach through “The Chemistry of Poisons” (11), an interdisciplinary elective featuring organic chemistry, biology, pharmacology, and toxicology content organized around the unifying theme of toxicological mechanisms. Student course feedback and retrospective survey results were consistent with knowledge and application-level learning and indicated a preference for interdisciplinary course design. Schroeder et al. describe a parallel effort implementing the MORE (Model, Observe, Reflect, Explain) pedagogy in a survey organic and biochemistry course for pre-health students (12), demonstrating that reconstruction can be implemented within existing laboratory infrastructure when supported by appropriate pedagogical frameworks.

The complementary three-stage evolution, with the effectiveness of each stage moderated by institutional context—faculty expertise, resources, student preparedness, and accreditation requirements—demonstrates that curriculum integration depth correlates positively with clinical knowledge transfer, but the optimal integration strategy is institution-specific rather than universal. This finding is consistent with the comparative analysis by Ma et al., who examined the integration of psychology and medicine from a Chinese perspective and found that innovative curriculum systems significantly improved student satisfaction (18), with curriculum design and teaching effect serving as significant predictors.

Table 2 provides a comparative summary of the three modular design strategies.

**Table 2:** Comparison of modular curriculum design strategies across key dimensions.

Strategy	Implementation complexity	Clinical transfer	Faculty requirement	Representative context
Linear module combination	Low	Moderate	Single-discipline sufficient	China, Russia
Clinical-topic spiral modules	High	High	Cross-disciplinary team	China (SJTU), US (Emory)
Flexible elective pathways	Medium	Variable	Multi-specialty coverage	US (Rochester)

### 4.1 Modular design strategies across institutional contexts

The comparative analysis of linear, spiral, and elective modular models across institutions in China, the United States, and Europe reveals consistent patterns in the contextual conditions under which each strategy produces improved learning outcomes. Clinical-topic spiral modules consistently outperform linear combinations in knowledge coherence and clinical transfer across diverse settings, but successful implementation is contingent on prior establishment of cross-disciplinary faculty collaboration mechanisms. The progressive relationship among these strategies—linear models serving as a prerequisite stepping-stone toward spiral and elective designs—suggests a developmental trajectory for institutional reform capacity.

Evidence from multi-year tracking studies provides the strongest available support for this progression. Zhong et al.'s three-year data from Shanghai Jiao Tong University demonstrates that spiral module effectiveness improves over successive cohorts as faculty gain experience and refine materials (2), a pattern consistent with a learning-curve interpretation of institutional reform capacity. Zhang et al. describe the integrated medical curriculum at Shanghai University School of Medicine, a newly established institution that implemented horizontal and vertical integration from inception rather than evolving through the three stages (4), demonstrating that institutional context—specifically the absence of legacy curricular structures—can accelerate reform progression.

In the United States, the Emory University curriculum reform described by McGill et al. represents a decade-long commitment to spiral integration at the departmental level (7), while Austin's “Chemistry of Poisons” represents a course-level reconstruction that can be implemented within existing departmental structures (11). The contrast between these two approaches illustrates the spectrum of reform scope, from departmental transformation to individual course innovation, and suggests that incremental course-level reforms may serve as pilot demonstrations that build institutional support for broader change. Gao and Qiao describe the exploration of integrated courses in materials subjects for promoting medicine-engineering integration at North China University of Science and Technology (19), using varying teaching methods including online-offline mixed teaching, case teaching, and flipped classroom, further illustrating the institutional diversity of reform approaches.

### 4.2 Ideological education integration as a culturally specific structural dimension

The integration of ideological and political education within medical chemistry courses represents a distinctive feature of Chinese curriculum reform with no direct Western counterpart, yet it addresses the universal educational challenge of professional value cultivation. Wang describes the exploration and practice of integrating ideological education into analytical chemistry teaching in medical colleges (6), demonstrating how topics such as pharmaceutical quality control, environmental pollutant analysis, and clinical laboratory standards can serve as vehicles for discussions of professional ethics, social responsibility, and scientific integrity.

The chemistry-medicine interdisciplinary context provides a natural vehicle for this integration through several mechanisms. Chemistry history education provides narratives of scientific discovery that illustrate values of perseverance, intellectual honesty, and commitment to human welfare. Pharmaceutical ethics discussions, anchored in cases such as the thalidomide tragedy or antibiotic resistance, connect chemical knowledge to the ethical dimensions of medical practice. Laboratory safety literacy, while ostensibly a technical competency, cultivates broader professional values of precision, responsibility, and concern for collective welfare. The key insight from the reform literature is that the effectiveness of this integration depends critically on instructional design quality: when ideological content emerges organically from chemical and medical content, students recognize it as a natural extension of professional formation rather than an externally imposed requirement.

The contrast between Chinese and Western approaches to professional identity formation within chemistry education reveals both divergent philosophical underpinnings and convergent practical strategies. Chinese medical education explicitly frames professional values as core competencies alongside technical skills, a position consistent with the CBME principle that competency encompasses more than knowledge and skills but diverges from Western models in its explicit ideological content. Western approaches tend to embed professional values within broader constructs such as professionalism, ethics, and social accountability, achieving similar educational goals through different conceptual framing. The transferable design principle—that discipline-specific content can serve as a vehicle for professional value cultivation—applies across contexts even when the specific values being cultivated differ.

## 5 Pedagogical innovation through contrastive complementarity of active learning methods

Teaching method innovation is the most extensively researched dimension of chemistry-medicine integration reform, with a literature that spans case-based learning (CBL), problem-based learning (PBL), flipped classroom, blended learning, and technology-enhanced instruction. This section provides a comparative analysis of these methods, distinguishing their applicability conditions, complementary roles, and the contextual factors that moderate their effectiveness.

### 5.1 Comparative effectiveness and contextual moderators of CBL and PBL

Case-based learning and problem-based learning, while often grouped together in the pedagogical literature, operate on fundamentally different instructional logics. CBL uses structured clinical cases to illustrate and apply previously taught chemical principles, providing a bridge between abstract knowledge and concrete clinical application. PBL begins with an unstructured clinical problem and requires students to identify the chemical knowledge needed to solve it, developing self-directed learning and cross-disciplinary reasoning skills (20).

The comparative effectiveness of these methods has been examined through meta-analytic approaches. Yan et al. conducted a Bayesian network meta-analysis of PBL and CBL teaching methods in clinical practical teaching (16), finding that the combination of PBL and CBL showed superior effectiveness across multiple outcomes including clinical thinking capacity, clinical practice capacity, knowledge understanding degree, literature reading ability, method satisfaction degree, learning efficiency, learning interest, practical skills examination scores, and theoretical knowledge examination scores. This finding—based on seven studies (five RCTs and two observational studies)—suggests that the two methods are complementary rather than competitive: CBL excels in structured clinical chemistry knowledge transfer, while PBL promotes deeper learning in complex cross-disciplinary problems. The closed-loop relationship between CBL (knowledge structuring) and PBL (knowledge application) emerges as a pedagogical design principle.

However, a critical evidence assessment reveals that reported effect sizes are systematically moderated by implementation factors. Aruna et al. compared CBL vs. didactic lectures in clinical biochemistry among first-year MBBS students (*n* = 60), finding that CBL produced superior outcomes for clinical reasoning skills but not for factual knowledge recall (21), with mean viva voce scores of 8.233 for CBL vs. 6.233 for didactic lectures on the assessment day, and 9.533 vs. 5.667 after 15 days. While these differences were statistically significant (*P* < 0.0001), the small sample and single-institution design limit generalizability. Eissa et al. describe a large-scale CBL application for teaching medical biochemistry to 1,490 students (22), reporting significantly improved posttest scores (*P* < 0.001) and positive student and faculty ratings, but the pre-post design without a concurrent control group limits causal attribution.

Class size, facilitator training, case quality, and assessment alignment emerge as the most consistently identified moderators of CBL and PBL effectiveness. Novack provides detailed guidance on designing cases for CBL in large medical school classes (23), emphasizing that effective cases must be authentic in their clinical detail, calibrated to student knowledge levels, and designed to surface the specific chemical principles that the curriculum targets. Sibbald et al. describe an integrated case-based training program adapted to public health workforce needs (24), highlighting the importance of aligning case complexity with learner readiness.

The implementation challenges of both methods are substantial and underappreciated in the literature. CBL requires the development of high-quality clinical cases that are both chemically accurate and clinically authentic, a task demanding collaboration between chemistry and clinical faculty that is rarely formalized in institutional structures. PBL requires even greater faculty facilitation skills, as instructors must guide student inquiry without reverting to direct instruction. Team-based learning (TBL), a related structured small-group approach, has been examined in a systematic review by Roossien et al., who found that most existing research examines TBL's effect on learning outcomes at the lower levels of Kirkpatrick's model (25), while higher-level outcomes such as communication skills and clinical reasoning remain underexplored. Xie et al. conducted a systematic review and meta-analysis of 33 studies (26), finding that TBL students exhibited significantly higher pre-test (SMD = 0.51, 95% CI: 0.11–0.92) and post-test scores (SMD = 0.96, 95% CI: 0.70–1.22), better retention (SMD = 1.03, 95% CI: 0.38–1.69), and higher satisfaction (SMD = 1.08, 95% CI: 0.87–1.29), but noted that students required more time for independent preparation.

### 5.2 Flipped classroom, blended learning, and technology-mediated instruction

The flipped classroom model has been widely adopted in medical chemistry education, with the three-stage design—pre-class knowledge delivery through micro-videos, in-class clinical case discussion, and post-class extended practice—serving as the most common implementation template. Li describes the application of the flipped classroom teaching mode in medical basic chemistry (27), using osmotic pressure of dilute solutions as a case example. The integration of micro-video technology with CBL represents a particularly promising innovation: Pan et al. investigated a micro-film CBL model in pediatrics education with 104 students (28), finding that the experimental group performed significantly better on student self-assessment (*P* < 0.05), satisfaction surveys (*P* < 0.05), and final examinations (80.02 ± 3.77 vs. 73.65 ± 3.69, *P* = 0.000).

International comparisons reveal consistent patterns in flipped classroom effectiveness. In South Korea, Kang and Kim found that blended learning with flipped classroom and TBL enhanced nursing students' knowledge (29), problem-solving ability, and learning satisfaction compared to traditional lectures. In Ethiopia, Mengesha et al. assessed flipped classroom effectiveness among undergraduate medical students at Gondar University through an interventional study (30). In the UK, Francis et al. adopted a flipped classroom approach for teaching molar calculations to biochemistry and genetics students (31), reporting positive student perception and significant improvement in summative assessment. In Hong Kong, Chan et al. conducted a randomized trial comparing flipped classroom case learning with traditional lecture-based learning in ophthalmology education (32), finding that the flipped model was effective for non-core specialty teaching where limited time and low student engagement are common challenges.

However, a critical reading of this evidence base reveals that the three-stage flipped design demonstrates modest but consistent learning gains across international contexts, with marginal benefits moderated by students' digital literacy and institutional platform infrastructure. The additive contribution of technology-mediated enhancements beyond the core flipped structure—virtual simulation, micro-video, AI-assisted instruction—remains weakly evidenced, with most studies comparing technology-enhanced flipped designs against traditional lectures rather than against non-technology-enhanced flipped designs. He constructed a “three-stage asynchronous” instructional mode of blended flipped classroom based on a mobile learning platform (33), finding that it improved academic performance, autonomy, collaborative inquiry, and self-efficacy, while Singhal et al. evaluated flipped classroom effectiveness in a pharmaceutical sciences course (34), finding modest improvements of 1%–5.5% in test averages with more notable differences in higher-level cognitive domains.

Li describes the implementation of a student-centered team-based learning method in a medicinal chemistry curriculum (35), organized around pre-class preparation, in-class team activities, and post-class consolidation. The results indicate that this approach improves student learning enthusiasm and initiative, with the team-based structure providing peer support that is particularly valuable for students who struggle with the abstract nature of chemical concepts. The student-centered design represents a shift from instructor-driven content delivery to learner-driven knowledge construction, broadly consistent with the CBME paradigm.

Beyond discipline-specific implementations, the design principles underlying innovative pedagogical strategies have been systematically examined in broader educational contexts. Suartama et al. (36) developed and validated a context-aware ubiquitous learning model based on case methods and team-based projects, using a research and development approach with validation involving five learning material experts, five learning media experts, and 162 students from Indonesian universities. The model integrates case-based learning within a technology-supported ubiquitous environment to guide the design, development, utilization, and evaluation of learning resources. Although the study was conducted in a teacher-training context rather than in medical chemistry education specifically, its instructional design framework—which emphasizes the convergence of case-method pedagogy, team-based collaborative projects, and context-aware technology-enhanced learning environments—offers transferable principles for medical chemistry education. The model's explicit attention to how learning media design can support competency development, student engagement, problem-solving, and collaboration mirrors the pedagogical objectives that medical chemistry educators seek when integrating CBL, PBL, and technology-mediated instruction (16, 22). In particular, the context-aware dimension of the model—where learning resources adapt to the learner's context—resonates with the adaptive and personalized learning approaches that are emerging as a frontier in medical education (34). While the direct applicability of the model to medical chemistry education requires empirical evaluation in that specific context, the validated design principles provide a structured framework that may inform the development of technology-enhanced interdisciplinary learning environments.

Table 3 provides a comparative analysis of the major pedagogical methods in medical chemistry education.

**Table 3:** Comparative analysis of pedagogical methods in medical chemistry education.

Method	Primary Mechanism	Key Advantage	Key Limitation	Evidence Strength
CBL	Structured clinical cases	Controlled content scope	Case development cost	Multiple RCTs + meta-analysis
PBL	Unstructured problem inquiry	Self-directed learning	High facilitation demand	Multiple RCTs + meta-analysis
CBL + PBL	Complementary sequencing	Complete learning cycle	Implementation complexity	Bayesian network meta-analysis
Flipped classroom	Pre-class content delivery	Maximized active learning	Digital literacy requirement	Multiple quasi-experimental studies
TBL	Collaborative knowledge construction	Peer support and engagement	Group dynamics management	Systematic review + meta-analysis

Figure 3 illustrates the contrastive complementarity between CBL and PBL, showing how they jointly form a complete learning cycle spanning knowledge acquisition, clinical application, and cross-disciplinary integration.

**Figure 3:**
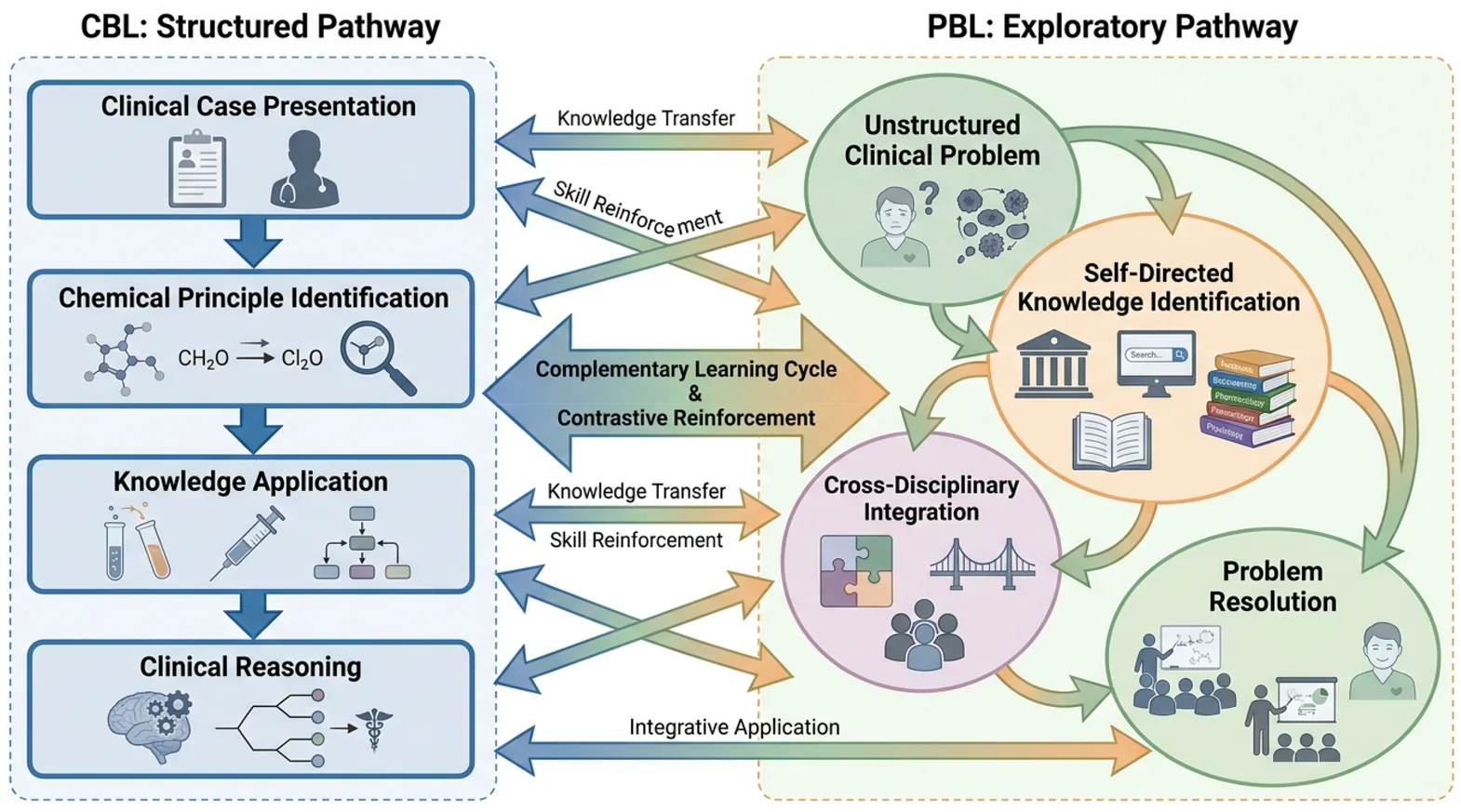
Contrastive complementarity between CBL and PBL in medical chemistry education.

## 6 Experimental teaching reform along a four-stage competency trajectory

Experimental teaching occupies a distinctive position in medical chemistry education as the primary site where chemical principles are translated into practical skills and where the integration of chemical and medical thinking becomes operational. The evolution of experimental teaching follows a four-stage trajectory that mirrors the broader competency-development ladder: confirmatory, comprehensive, inquiry-based, and interdisciplinary innovative.

Confirmatory experiments, in which students follow prescribed procedures to verify known chemical principles, remain the most common format globally but contribute minimally to clinical reasoning or research competency. Han identifies the limitations of traditional confirmatory experiments—unoriginal content, simplistic results (37), uniform reports, low student engagement, and inadequate development of independent learning skills—and argues that the advancement of biochemical theory and technology has made innovation capacity a priority for universities, research institutes, hospitals, and enterprises alike. This recognition has driven the progressive advancement toward higher-stage experimental designs across multiple national contexts.

Comprehensive experiments integrate multiple techniques within a single session, providing a more holistic picture of chemical investigation. Inquiry-based experiments require students to design or modify procedures to answer open-ended questions, developing scientific reasoning skills. Interdisciplinary innovative experiments represent the highest level, combining chemical synthesis, structural characterization, and biological activity evaluation within a single integrated design. Ma describes the experimental reform of medical functional science based on technological innovation (38), reporting the development of an integrated experimental course combining pharmacology, physiology, and pathophysiology experiments that created a rich, interactive teaching environment significantly improving teaching effectiveness, quality, and efficiency. Hong reports on the reform and practice of experimental teaching in the foundation of clinical laboratory medicine (39), demonstrating that reorganizing content, reforming methods, and establishing an inspection and assessment system can remarkably enhance student experimental skills, clinical post skills, and teacher enthusiasm.

The integrated design experiment model—combining chemical synthesis, structural characterization, and biological activity evaluation—has emerged as the core breakthrough in experimental teaching reform. Li describes the reform of precise teaching for experiments in medical laboratory technology (40), implementing precise stratification of experimental projects and knowledge points, precise teaching activity design, and precise evaluation system construction. Guided by precision medicine principles, this reform strengthened standardization and precision training of basic skills, better preparing students for the technical and innovative demands of modern clinical laboratory practice. However, the evidence for integrated designs, while consistently positive, derives from single-institution implementations with modest sample sizes, and the scalability of these designs is limited by faculty interdisciplinary expertise and specialized equipment requirements.

### 6.1 Integrated design experiments as the core breakthrough

The integrated synthesis-characterization-bioactivity experimental design achieves organic fusion of chemical operation skills and medical research thinking by aligning experimental structure with authentic scientific practice. This alignment explains why integrated designs produce stronger learning outcomes than confirmatory experiments: they engage students in the same practices—hypothesis formation, experimental design, data interpretation, and cross-disciplinary reasoning—that characterize professional scientific and clinical work. Liang describes the teaching reform of general chemistry experiments oriented by innovation ability training (41), restructuring assessment so that 50% of the grade derives from experimental reports (including online preparatory tests and post-laboratory paper-style reports) and 50% from experimental operation examinations, effectively integrating knowledge acquisition with practical skill demonstration.

Yang reports on measures to promote the teaching reform of biochemical laboratories using national experimental skills competitions (42), illustrating an additional motivational mechanism through competition-driven reform. The diverse institutional contexts documented in the literature—from Nankai University's top-talent cultivation program (43) to Guangzhou University of Chinese Medicine's four-level progressive training model (44)—demonstrate that experimental teaching reform is being pursued through multiple pathways, with the specific reform strategy calibrated to institutional resources, faculty expertise, and student characteristics.

Despite these advances, the evidence consistently indicates that higher-stage reforms encounter diminishing returns constrained by faculty cross-disciplinary capacity and experimental platform integration. The faculty expertise required to design and supervise interdisciplinary innovative experiments is fundamentally different from that required for confirmatory experiments, and current faculty development models, which typically train chemists and clinicians in separate disciplinary silos, are poorly suited to producing this expertise.

### 6.2 Virtual simulation and hybrid models across resource contexts

Virtual simulation and online-offline hybrid experimental models have emerged as significant innovations, addressing traditional limitations in reagent consumption, hazardous waste disposal, equipment availability, and safety constraints. The advantages of virtual simulation can be characterized along three dimensions: low-cost trial-and-error capability allowing students to explore experimental variations without consuming reagents or generating waste; visual interaction capability enabling students to observe molecular-level phenomena invisible in traditional experiments, such as reaction mechanisms, molecular docking, and spectroscopic transitions; and safe operation capability permitting engagement with hazardous reagents or conditions excluded from undergraduate laboratories on safety grounds.

International comparisons reveal important patterns in how virtual simulation adoption varies by resource context. In the United States, Qin et al. describe a wireless, PC-less portable virtual reality chemistry lab applied in an undergraduate biochemistry class with over 200 students at Harvard University (45), demonstrating that VR technology can be deployed at scale in high-resource settings. Karayilan et al. reassessed undergraduate polymer chemistry laboratory experiments for virtual learning environments, noting that virtual labs can complement face-to-face laboratories and offer a cost-efficient (46), safe, and environmentally sustainable alternative. In Indonesia, Soraya et al. examined the benefits and challenges of implementing virtual laboratory simulations for medical biochemistry, reporting increased motivation and the ability to master content through repetition (47), but also identifying technical and language barriers in a cohort of non-native English speakers.

In lower-resource settings, virtual simulation addresses infrastructure and safety constraints most effectively. Chernov describes physical chemistry experiment teaching reform under innovation ability training (48), while Lai describes instructional design for optimizing biochemical chemistry education using basic medical knowledge (49), demonstrating how virtual pre-laboratory preparation can improve the efficiency and safety of subsequent physical laboratory work. However, evidence consistently indicates that virtual simulation functions best as a pre-laboratory complement to physical experimentation rather than a replacement. Students who complete virtual simulations before entering the physical laboratory arrive with better conceptual understanding of experimental principles, more accurate mental models of procedural steps, and greater awareness of safety considerations, but the development of physical manipulative skills, chemical intuition, and troubleshooting ability requires hands-on experience.

The environmental dimension of experimental teaching reform has received increasing attention. Ma discusses the reform of chemistry laboratory teaching under the construction of a green ecological environment (50), proposing greening strategies including microscale experiments, solvent substitution, and waste minimization. This environmental consciousness aligns with the broader sustainability agenda in chemical education (5) and provides an additional dimension of professional value education within the experimental curriculum.

Table 4 compares the four experimental teaching stages across key implementation dimensions.

**Table 4:** Characteristics of the four-stage experimental teaching trajectory.

Stage	Student role	Learning outcome	Faculty requirement	Resource need
Confirmatory	Procedure follower	Factual verification	Basic chemistry	Standard equipment
Comprehensive	Multi-technique operator	Technique integration	Multi-technique expertise	Multiple instrument types
Inquiry-based	Procedure designer	Scientific reasoning	Research mentorship	Flexible equipment access
Interdisciplinary innovative	Independent investigator	Clinical-scientific fusion	Dual chemistry-clinical expertise	Integrated platform

## 7 Teaching effectiveness evaluation and a multi-level assessment framework

The evaluation of teaching effectiveness in chemistry-medicine integration reform represents both a critical research domain and a significant methodological challenge. Current evaluation practices are characterized by three systemic limitations: fragmented instruments that lack cross-context validity, predominantly small single-center samples that limit statistical power and generalizability, and short-term tracking that cannot capture longitudinal competency development.

### 7.1 Design and validation of composite evaluation instruments

The design of evaluation instruments for chemistry-medicine integration must address the multidimensional nature of the learning outcomes involved. Traditional approaches relying primarily on written examinations of chemical knowledge capture only the lowest level of the competency hierarchy. Composite evaluation tools that integrate knowledge mastery, clinical reasoning, and practical skills are essential for measuring the distinctive outcomes of interdisciplinary teaching reform.

Eissa et al., in their large-scale CBL study, employed a multi-dimensional evaluation approach combining student self-assessment questionnaires, satisfaction surveys, and final examinations (22). Yan et al. examined outcomes across nine dimensions in their Bayesian network meta-analysis (16). Mendes et al. describe the evaluation of competencies developed in a medicinal chemistry discipline through the creation of review texts (51), representing an authentic assessment approach that evaluates students' ability to synthesize and communicate interdisciplinary knowledge. This approach aligns with the CBME paradigm but presents challenges in standardization and scalability.

Composite evaluation instruments integrating knowledge mastery, clinical reasoning, and practical skills are essential, yet the proliferation of study-specific instruments without shared validation frameworks prevents meaningful cross-study comparison and evidence accumulation. Each reform initiative develops its own measures, reports its own outcomes, and the field lacks the infrastructure for cumulative knowledge building. The development of shared instrument banks with documented validity evidence, standardized outcome taxonomies, and longitudinal tracking mechanisms would enable the field to move beyond its current fragmented state.

### 7.2 International comparison, methodological diversity, and evidence gaps

International comparison reveals both convergence and divergence in evaluation approaches. In the United States, Halman et al. explored the use of an entrustability rating scale within an OSCE progress test for internal medicine residents (52), generating highly reliable ratings (Cronbach's alpha indicating high inter-station reliability) that discriminated between learners at different training levels. In the UK, Kumaravel et al. developed and evaluated a spiral model of assessing evidence-based medicine competency using OSCEs in undergraduate medical education (53), demonstrating the feasibility of authentic assessment of EBM performance in high-stakes settings. In India, Muralidharan et al. assessed the feasibility and utility of OSCE as an end-of-internship assessment over six years (*n* = 469) (54), finding it to be a comprehensive tool that underscored the importance of multi-modal evaluation strategies while noting substantial resource requirements (528–843 faculty-hours per batch).

The predominance of single-center small-sample studies in the current evidence base represents a methodological limitation for generalizability rather than an evidence-quality hierarchy. High-quality educational research employs diverse methodologies beyond RCTs, including well-designed quasi-experimental studies, mixed-methods designs, and longitudinal tracking, each providing valid complementary evidence. Sterpu et al. conducted a cost-analysis of TBL vs. small group interactive learning (55), finding that TBL was more costly primarily due to implementation expenses for faculty training and electronic platforms, while Jabeen et al. found that TBL improved knowledge acquisition independent of student ratings of TBL sessions (56).

The methodological observation that evidence concentrates in single-center small-sample studies is discussed here as a feature of the current literature that limits generalizability, not as a quality judgment about individual studies. Coordinated investment in shared evaluation infrastructure—multi-center study networks, shared instrument banks with documented validity evidence, standardized outcome taxonomies, and longitudinal tracking mechanisms—would enable the field to move from isolated case reports toward cumulative science. The development of these resources, rather than the adoption of any single study design, represents the most urgent methodological priority for the field.

Figure 4 illustrates the proposed multi-level assessment framework, showing four assessment levels with their representative instruments.

**Figure 4:**
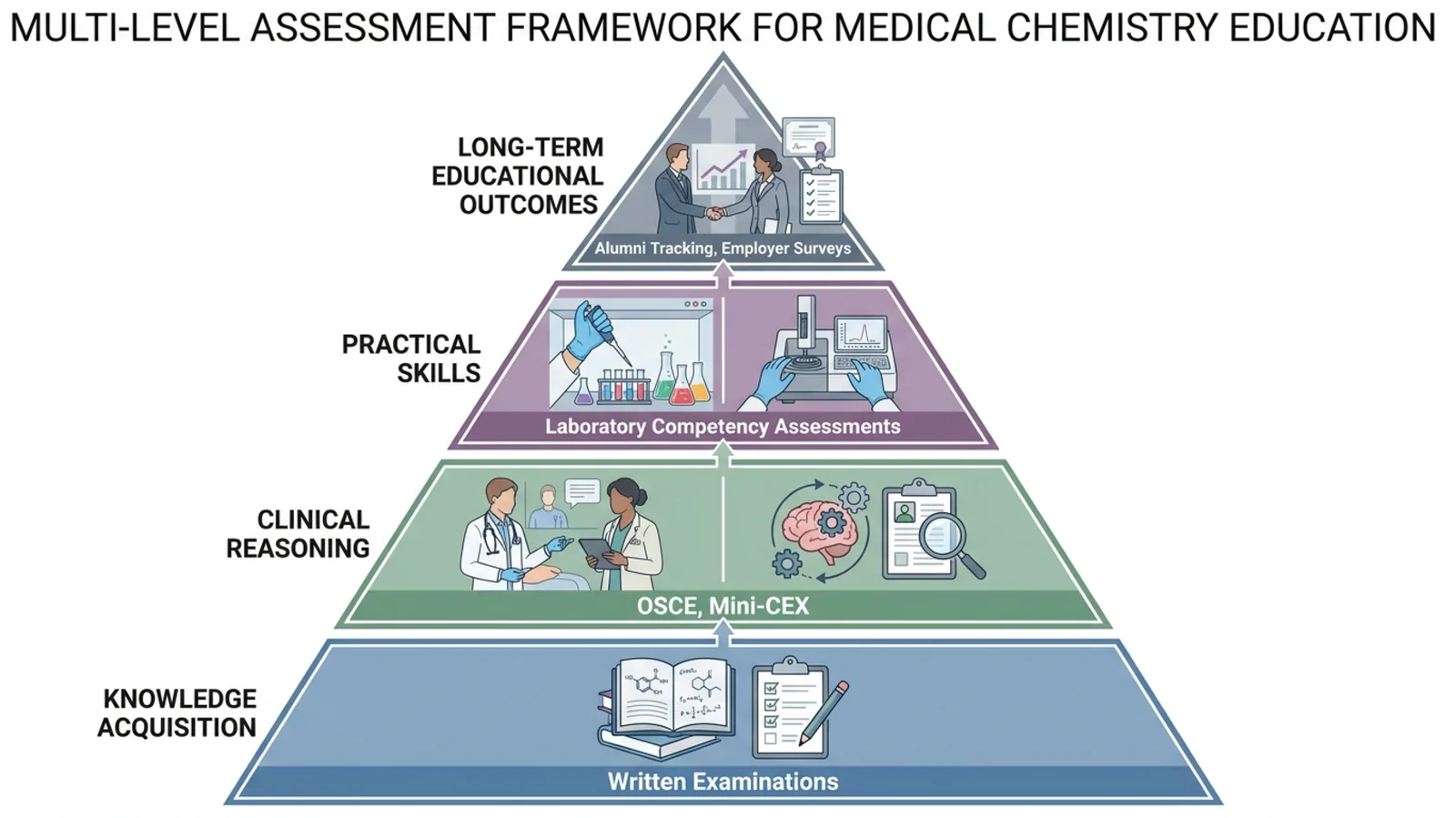
Multi-level assessment framework for chemistry-medicine integration outcomes.

## 8 Conclusions and future directions

This review has applied the five-dimensional analytical framework—with the traditional discipline-based teaching model as the comparison baseline—to systematically examine research progress on chemistry-medicine interdisciplinary teaching reform from 2016 to 2025. The analysis, grounded in 105 studies identified through a reproducible search protocol, reveals that the field has progressed from isolated exploration to systematic construction, with theoretical foundations, curriculum models, pedagogical methods, experimental designs, and evaluation approaches all advancing substantially. However, three persistent bottlenecks constrain further progress.

First, cross-disciplinary faculty development remains the most critical structural barrier. The implementation of integrated curricula, problem-driven teaching methods, and interdisciplinary experimental designs requires faculty who are fluent in both chemical and clinical reasoning, yet current faculty development models, which train chemists and clinicians in separate disciplinary silos, are inadequate for this demand. Multi-institutional faculty development programs for interdisciplinary pedagogy and institutional mechanisms for cross-disciplinary collaboration represent urgent infrastructure priorities.

Second, resource integration mechanisms are underdeveloped. The development of high-quality clinical cases, virtual simulation platforms, integrated experimental modules, and validated evaluation instruments requires sustained investment exceeding the capacity of individual institutions. Open-access clinical case repositories tailored to medical chemistry, shared virtual simulation platforms, and multi-institutional consortia could address this gap but require coordination mechanisms not yet established.

Third, evaluation standard unification is essential for the field to progress from isolated case reports to cumulative knowledge. The current fragmentation of evaluation instruments, outcome measures, and study designs makes cross-study comparison and the establishment of comparative effectiveness for different reform strategies impossible. The development of a consensus outcome taxonomy, validated core instrument set, and multi-center study infrastructure would enable evidence accumulation that currently eludes the field.

Two breakthrough directions are identified, though their evidence base is nascent. AI-empowered precision teaching, including adaptive learning systems, intelligent tutoring, and learning analytics, represents a technological frontier whose potential remains largely untested through rigorous evaluation. The systematic alignment of medical chemistry reform with New Medical Education construction frameworks provides an institutional and policy pathway for scaling reform from isolated exemplars to systemic practice, but this alignment must be accompanied by investment in the evaluation infrastructure needed to generate credible evidence of effectiveness.

Specific, actionable recommendations emerging from this review include: (1) establishing shared evaluation instrument banks with documented validity evidence across contexts; (2) creating multi-institutional faculty development programs for interdisciplinary pedagogy; (3) developing open-access clinical case repositories tailored to medical chemistry education; and (4) initiating longitudinal tracking studies that follow graduates into clinical practice to assess long-term reform impact. These recommendations are offered with the recognition that the evidence supporting them derives primarily from single-institution studies, and their effectiveness requires multi-center validation.

The five-dimensional evaluation framework established in this review is intended to serve as a comparative coordinate system for future reform design and implementation. By specifying the dimensions along which reform pathways can be evaluated and the baseline against which they can be compared, the framework supports the systematic accumulation of evidence that the field currently lacks. The ultimate measure of success for chemistry-medicine integration reform is the improvement in clinical competency that it enables.

## 9 Comprehensive evidence summary tables

This section presents four comprehensive evidence summary tables that synthesize the reviewed literature across key analytical dimensions, providing a structured overview that enables readers to assess the evidence base independently.

Table 5 summarizes studies by educational intervention type, student population, study design, sample size, outcome measures, key findings, and limitations. The table reveals several patterns in the evidence base: CBL and PBL interventions are the most frequently studied, with meta-analytic evidence providing the strongest support for combined approaches; flipped classroom studies span the widest geographic range but exhibit the greatest heterogeneity in study design quality; and virtual simulation studies concentrate in resource-limited settings where laboratory infrastructure constraints are most acute.

**Table 5:** Evidence summary by educational intervention type.

Intervention	Population	Design	Sample	Key finding	Limitation
CBL	Medical biochemistry	Pre-post	1,490	Significant posttest improvement	No control group
PBL + CBL combined	Clinical teaching	Bayesian NMA	7 studies	Superior across 9 outcomes	Only 5 RCTs included
Flipped classroom	Pharmacy	Quasi-experimental	137	Significant performance gain	Single institution
TBL	Medical education	Meta-analysis	33 studies	SMD = 0.96 post-test	Heterogeneous designs
Virtual simulation	Medical biochemistry	Pre-post	271	Increased motivation	Technical barriers
Integrated experiments	Medical lab technology	Descriptive	N/R	Improved skill training	No comparison data

The evidence base across all intervention types is characterized by a predominance of single-institution studies with sample sizes typically below 200 participants, and short-term outcome measures (end-of-course examinations, student satisfaction surveys) rather than long-term clinical competency tracking. These methodological features, while not indicative of individual study quality, collectively represent the most significant barrier to generalizable conclusions in the field.

The multi-level assessment framework proposed in this review, operationalized through the instrument-outcome matrix linking knowledge, clinical reasoning, practical skills, and long-term outcomes, is intended to address the evaluation fragmentation that currently prevents evidence accumulation. Table 6 presents this matrix, mapping each assessment level to specific instruments, target outcomes, and the strength of existing validity evidence.

**Table 6:** Multi-level assessment framework: instruments, outcomes, and validity evidence.

Assessment level	Representative instruments	Target outcome	Validity evidence
Knowledge acquisition	Standardized examinations, MCQ tests	Factual recall, concept understanding	High (established instruments)
Clinical reasoning	OSCE, Mini-CEX, case-based assessments	Diagnostic reasoning, knowledge application	Moderate (context-dependent)
Practical skills	Laboratory competency assessments, portfolios	Technical proficiency, safety practices	Variable (institution-specific)
Long-term outcomes	Alumni tracking, employer surveys, patient outcomes	Clinical practice quality, career development	Low (few studies available)

Table 7 identifies key research gaps organized by framework dimension, current status, and proposed methodological approaches. The table underscores that while pedagogical innovation is the most extensively researched dimension, the evidence base remains weakest for long-term outcome evaluation and cross-disciplinary faculty development.

**Table 7:** Research gaps and methodological priorities by framework dimension.

Dimension	Key gap	Current status	Priority approach
Driving forces	Cross-national comparative motivation studies	Few studies available	Multi-country survey research
Curriculum restructuring	Long-term knowledge retention evidence	Single-institution tracking only	Multi-institution longitudinal designs
Pedagogical innovation	Implementation fidelity measurement	Heterogeneous reporting	Standardized fidelity frameworks
Practical translation	Scalability beyond pilot implementations	Mostly small-scale studies	Multi-center replication studies
Outcome evaluation	Clinical practice impact assessment	Almost no evidence available	Graduate tracking + employer surveys
